# Foreword of an American Balneology

**Published:** 1916-10

**Authors:** Felix Von Oefele

**Affiliations:** New York


### BUFFALO MEDICAL JOURNAL

Yearly Volume 72 OCTOBER, 1916 Number 3

### ORIGINAL ARTICLES

The right is reserved to decline papers not dealing with practical medical and surgical subjects, and such as might offend or fail to interest readers. Contributors are solely responsible for opinions, methods of expression and revision of proof.

### Foreword of An American Balneology.

DR. FELIX VON OEFELE, New York.

The American physician is less acquainted with the American mineral springs than with the European ones. This seems astonishing in some way, since official research and official promotion is done by the Federal government in liberal amount for the basis of American balneology. The literature of American balneology is the proof of it.

The ownership influences the development. European mineral springs with an old history are owned by states or townships. The newer European springs are owned in large percentage by individual private people. A considerable number of American mineral springs became public property of the states or the federal government only in the last century. This development is quite different from Europe. The few older public American mineral springs are overshadowed by private springs of their vicinity. A strong majority of the modern American mineral springs are still individually owned. The principal American scientific balneological research kept the close relationship to the older public or federal mineral springs as substitutes of more famous European mineral springs.

The kindred advertisement of the American mineral springs stays either on a very low level or is too exaggerated in fantastic claims. Both procedures tend to evade the contact with the medical profession.

There exists neither a scientific standard nor a critical view in the recommendation of American springs. Every American mineral spring owner knows and believes in his own spring only. He claims it a panacea and looks with a supercilious countenance upon every other spring. The therapeutic claims are based on some letters of laymen with political or social pull, who had some reason at that time to flatter the owner or his spring. The resident physicians are not consulted and are without any interest for the newly detected mineral spring. For further promotion, joint steps are unknown to the private owners of American springs. Baederalmanach or official compilations are lacking for the reference of physicians.

Furthermore, scientific medicine is too little interested in balneology. The scientific parts of professional periodicals contain less balneological matter than they did a century ago, and less than the European journals. It should be of more interest for the medical reader that the professional journals in the spring time bring extracts from publications concerning mineral springs with critical views particularly. There is a demand for information.

The enumeration of American balneological literature will show few books of Anderson, Crook, etc. But they cannot be compared with European scientific text books. For the greater part the American books are a mosaic, built from the unscientific answers of spring owners. And this can never be complete. The scientific collector commonly fails, expecting answers from spring owners. The European scientific advertisement of mineral springs has some effective schedule. One is the individual scientific booklet written by every average resident physician of the resort town. I have seen very few similar American booklets, for instance a booklet of a physician of Virginia Hot Springs in regard to gout. The large amount of similar European professional booklets furnishes the balneological compilator sufficient data.

The first need for an American balneologist is to be a more efficient collector than his European colleague. He has to collect first the names of the claimed mineral springs of 200 years, the names of the owners, detailed data of the location in geographical, physical and geological regard. The second are historical data; the third, other special circumstances. The exact geographical location is very important for identification of springs. It is still in doubt as to which springs of Saratoga County were used by Sir Johnson. Also the identification of other old mentioned springs is still doubtful on account of the absence of correct location. Street, township, county, state are often necessary.

The special difficulty of the collector starts by the average short efficiency of American mineral springs. Between 80 to 90% are again out of business in an average of four years. The inquirer cannot collect the names of the springs quick enough for his files during the real business period of the springs.

Many of the remaining springs refuse to answer any letter of a professional man. Either the fairness of the spring is insufficient, or the spring owners are afraid to emit data for the benefit of a new competitor seeking information. Some of the answers feign to be kind by sending wordy phrases without any real information.

The few remaining informations are in a high percentage not trustworthy or evidently untrue. It is a common event to receive as an answer an analysis which was really published in a local paper some decades ago, but is the analysis of some neighboring spring out of business.

The average American physician has therefore a reason why he does not trust to the springs of his own country. He prefers the trustworthy European mineral springs. A mineral spring cure of any serious sickness was understood before the war to be a European one.

There are some advantages to a European cure. A trip on the sea is often an adjutory therapeutic agent and is often an assistant remedy. In other cases a trip on the sea is contra-indicated. A visit to Europe is often undesirable or, more often wholly impossible. If the degree of common culture would rise then there would be a larger percentage of the population visiting the mineral springs of the vicinity for a few weeks in summer.

There is still another historical reason for the prejudice of American physicians against the American springs. The original investigators of the American springs were the Indians. Their medicine men are considered mere quacks as compared to the scientific physicians of the European settlers.

The oldest history of Ballston Spa has been proved. The European physicians of New York had no therapeutic success in the gout of the governor. Indian medicine men of the Iroquois recommended the use of the mineral waters of Saratoga county and with success. The laymen, i.e. Indian treatment there posed as successfully superior to the academic medicine. Similar cases may have happened very often, but have not been recorded in history. The farmer laymen received some knowledge concerning the local mineral springs and delivered it down from generation to generation. This kind of American balneology survived below the covering surface of the recording sciences.

Even now if sick people no longer expect any more help from the regular physician, another layman persuades them to a trial of a domestic spring in an old shape of advice. That is felt as an offense to the profession and ethics of the

American physicians. Therefore the administered European spring became ethical, but the domestic American spring was quackery. But in some cases the real value can be exactly inverted. Especially in the home of sick people must the use of mineral waters be preferred to the use of domestic water.

An artificial mineral water never reaches the value of a natural one. The mutual arrangement of the atoms of the many elements present is different. The different electric conductibility shows the difference. These differences are of physical nature. It is understood that continued strong physical influences can change the physical condition. A natural mineral water is less active when it has been carried a few miles, than drunk directly from the spring. A long transportation over the ocean spoils every mineral water more or less. On this account American mineral waters are preferable for home use. But European mineral waters are more readily obtainable and cheaper in the large American cities than American ones.

For the construction of a useful American balneology, a complete collection of printed information is very important. Every little contribution approaches this completeness. Very important material may now be obtainable, but may be lost forever within a short time. For Saratoga, John M. Clarke states that the older notes of thrillings are incomplete, inaccessible or entirely lost. I learned myself how this happened. I inspired the artesian wells of Lower Bavary. I had collected notes concerning 150 such artesian wells in a small district of bracky Meiokaen deposit, which contained a marine sand stratum bearing water. I have published nothing of this collection and lost it later on changing my domicile. There I found many geological faults distant from 500 to 2,000 meters one from the other, with ascensions or descensions of the sand bed on the other side from 20 to 100 meters and more. It was verified by other stratigraphic statements and by the present or absent influence of water pressure of different wells. Some times two distant wells communicated.

326 East 58th Street.

---

**Haemo-Lymph Nodes.** Ed. Retterer, Soc. de Biol., Mch 18, states that the center of lymph nodes is a focus of cellular proliferation. In the young adult, iron is not indicated by the ferrocyanid test. But, as the tissue of the node develops, its cytoplasm tends to liquify and the nuclei which gain the periphery of the gland, enrich themselves with iron, detectable by this test. The iron seems to be available for the production of haemoglobin, the lymph node, like the spleen is both a magazine and an accumulator of iron.

